# A randomised controlled trial evaluating internal limiting membrane peeling forceps in macular hole surgery

**DOI:** 10.1007/s00417-022-05932-y

**Published:** 2022-12-13

**Authors:** Mariantonia Ferrara, Antonio Rivera-Real, Roxane J. Hillier, Maged Habib, Mustafa R. Kadhim, Maria T. Sandinha, Katie Curran, Alyson Muldrew, David H. W. Steel

**Affiliations:** 1 Sunderland Eye Infirmary, Queen Alexandra Road, Sunderland, SR2 9HP UK; 2 Bioscience Institute, Newcastle University, Newcastle Upon Tyne, UK; 3 Newcastle Eye Centre, Royal Victoria Infirmary, Queen Victoria Road, Newcastle Upon Tyne, UK; 4 Translational and Clinical Research Institute, Newcastle University, Newcastle Upon Tyne, UK; 5 St. Paul’s Eye Unit, The Royal Liverpool University Hospital, Liverpool, UK; 6 Department of Eye and Vision Science, Institute of Ageing & Chronic Disease, University of Liverpool, Liverpool, UK; 7 Centre for Public Health, Queen’s University of Belfast, Belfast, UK

**Keywords:** Dissociated optic nerve fibre layer lesions, Full-thickness macular hole, Inner retina defects, Internal limiting membrane, Internal limiting membrane forceps, Proof of concept randomised controlled trial, Swelling of arcuate nerve fibre layer lesion

## Abstract

**Purpose:**

To assess study design and a range of anatomical and functional changes after internal limiting membrane (ILM) peeling using forceps developed for atraumatic ILM pick-up compared to standard forceps.

**Methods:**

We conducted a masked proof-of concept randomised controlled trial (RCT) on 65 patients who underwent ILM peeling for idiopathic full-thickness macular hole (FTMH) using etched-tip forceps (etched-tip group, 33 eyes) compared to standard ILM forceps (smooth-tip group, 32 eyes). Patients were assessed preoperatively, 3 weeks, 3 and 6 months postoperatively.

**Results:**

The primary closure rate was 95.4%. There was no statistically significant difference between the groups in terms of final visual acuity (66.9 vs 70.9 ETDRS letters, *p* = 0.13), difference of visual field mean deviation (1.32 vs 1.14 decibels), and number of eyes with pick-up-related retinal haemorrhages (16% vs 16%, *p* = 0.96), swelling of arcuate nerve fibre layer lesions (63% vs 55%, *p* = 0.54), number of dissociated optic nerve fibre layer lesions (31.4 vs 41.0, *p* = 0.16), nor inner retina defects (37% vs 22%, *p* = 0.17). Similar changes in inner retinal volumes were detected in all 9 sectors of an ETDRS grid except for a trend (*p* = 0.06) towards a lower reduction in the inferior inner sector in the etched-tip group.

**Conclusions:**

The study was successfully completed with masking maintained and a low risk of bias. Multiple endpoints relating to ILM peeling were assessed, and estimates were provided that can be used for future studies. Although the study was not powered to assess any specific endpoint, the anatomical and functional outcomes assessed did not significantly differ.

**Supplementary Information:**

The online version contains supplementary material available at 10.1007/s00417-022-05932-y.


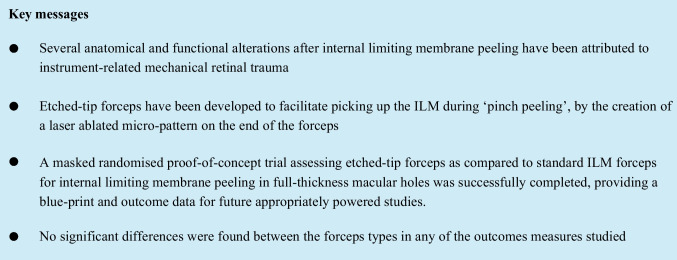


## Introduction

Internal limiting membrane (ILM) peeling has become an integral part of the surgical repair of primary idiopathic full-thickness macular holes (iFTMH) and is a common step in a variety of different vitreoretinal procedures, offering several advantages [1]. These include the release of peri-foveal tangential traction and an increase in retinal compliance resulting in higher rates of hole closure, as well as the removal of the scaffold for recurrent epiretinal membrane (ERM) formation [1–3]. However, various retinal anatomical changes have been observed after ILM peeling with potentially adverse effects. Among them, a dissociated optic nerve fibre layer (DONFL) appearance is considered a consequence of ILM peeling itself [4], whilst subacute swelling of the arcuate nerve fibre layer (SANFL) is thought to be a consequence of instrument-related mechanical trauma to the retinal nerve fibre layer (RNFL) [5, 6]. To start ILM peeling, the creation of the initial ILM flap is commonly performed using specifically designed vitreoretinal ILM forceps and the ‘pinch peeling’ technique [7]. Intraoperative forceps grasp site defects at the ILM pick-up points appear to be associated with SANFL, potentially leading to subsequent late NFL thinning, paracentral scotomata and reduced vision [6–9].

**Fig. 1:**
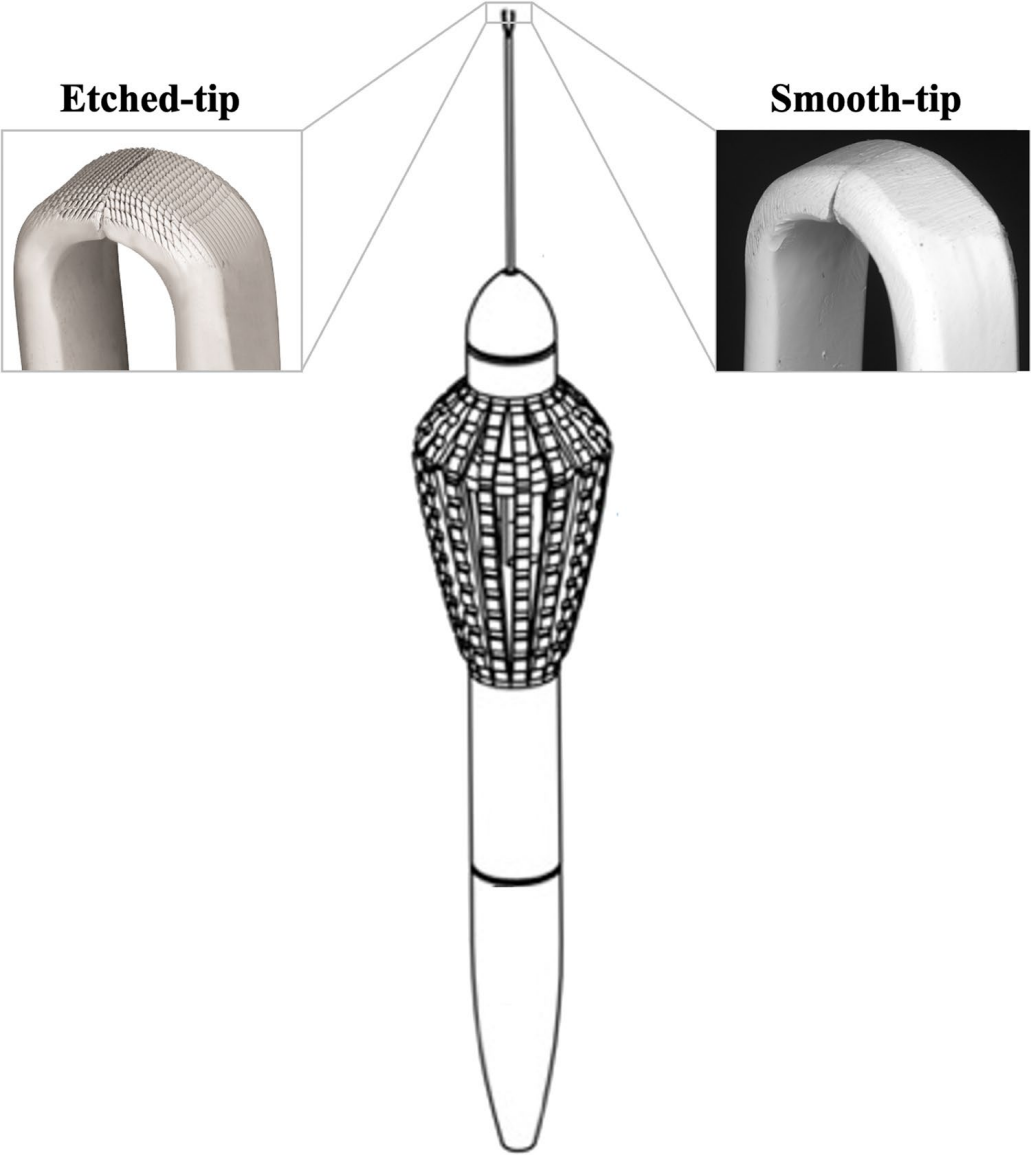
Schematic diagram of etched-tip and standard ILM forceps. The forceps differ only for the tip that in etched-tip model has a specific pattern of 10 × 10 × 5 micron teeth (created on the forceps surface by laser ablation) pointed towards the grasping edge at 30 degrees

Etched-tip forceps have been developed to facilitate picking up the ILM atraumatically, by the creation of a laser-ablated micro-pattern on the end of the forceps (Fig. 1). This design aims to increase friction across the forceps tips to engage the ILM and reduce the downward force on the retina required, ultimately minimizing iatrogenic inner retinal trauma [10].

Multiple parameters need to be considered to evaluate potential forceps-related iatrogenic trauma, with their extent, methodology of assessment and variability ill-defined and no clear parameters to guide sample size calculation. In this regard, feasibility and proof-of-concept studies play a crucial role in investigating this area, representing a key first step in assessing specific study designs and endpoints with the intent to use them in subsequent larger-scale trials [11].

We therefore conducted a proof-of-concept randomised controlled trial (RCT) of patients undergoing conventional ILM peeling for primary iFTMH using standard smooth-tip ILM peeling forceps (Alcon Grieshaber REVOLUTION DSP ILM forceps, Alcon, Fort Worth, TX, US) as compared to etched-tip ILM forceps (Alcon Grieshaber FINESSE SHARKSKIN ILM forceps). We assessed the appropriateness of the trial design including masking and multiple exploratory functional and anatomic outcomes.

## Methods

This masked randomised controlled feasibility trial was registered on the ISRCTN registry (reference 70,557,873), and the protocol is available at https://eprints.ncl.ac.uk/280333. Surgeries were performed by 5 experienced fellowship-trained vitreoretinal surgeons in 3 tertiary ophthalmology centres in the UK: Sunderland Eye Infirmary, Sunderland (DHWS, MH), Royal Victoria Infirmary, Newcastle upon Tyne (RJH, MRK) and Royal Liverpool University Hospital, Liverpool (MTS). All surgeons had independently performed at least 70 FTMH surgeries with ILM peeling prior to the study start. UK multicentre ethical approval was obtained (North of Scotland Research Ethics Committee reference 19/NS/0124). After a comprehensive discussion, all patients signed a written consent form.

### Sample size

As a proof-of-concept study, the sample size was set as 60, based on the methodology of Viechtbauer et al. [12]. It was not powered to assess any set endpoint, and a range of exploratory endpoints were analysed. We aimed to recruit 66 patients, to allow for 10% of cases lost to follow-up (FU). Each surgeon performed at least 12 surgeries.

### Recruitment criteria

We included patients over 50 years affected by iFTMH of any size and less than 12-month duration. We excluded patients in whom ILM peeling was not planned, secondary FTMH, previously vitrectomized eyes, pre-existing significant macular disease (early/intermediate AMD allowed), glaucoma, optic nerve disease, diabetic retinopathy worse than background retinopathy, uncontrolled intraocular inflammation, high myopia, amblyopia and conditions hindering visual fields (VFs) or imaging.

### Participants, randomisation and masking

Patients were randomised into two groups based on the forceps used for the ILM peeling: etched-tip ILM forceps in group ‘etched-tip’ and in group ‘smooth-tip’. Randomisation to study intervention was carried out by research staff using online randomisation, with a block size of 2 (REDCap, https://projectredcap.org/about/) immediately prior to surgery, with stratification by surgeon, hole size (≤ and > 400 μm in minimum linear diameter) and duration of symptoms (< 6 m and 6–12 m). Surgeons were masked to the forceps used. Both forceps had the same external appearance, and all packaging was removed to maintain surgeon masking. The etchings on the tip are not visible without magnification and, being on the distal tips, are not visible during peeling. Surgeons were asked to avoid deliberately viewing the distal tips prior to use under magnification.

### Surgical procedure

Surgery was standardised with 23- or 25-gauge transconjunctival pars plana vitrectomy with posterior hyaloid face separation, if not pre-existing. All phakic patients underwent combined cataract surgery. After core and peripheral vitrectomy, ILM staining was performed with heavy brilliant blue G (BBG) 0.025% for 30-s contact time. All surgeons used pinch peeling as their preferred ILM peeling initiation technique. After the air-fluid exchange, 16% C2F6 was used as tamponade, and patients were instructed to position face-down for 3 days then non supine for 7 days. All surgeries were recorded.

### Surgery evaluation

Immediately following surgery, surgeons were asked to guess the type of forceps allocated, disclose any unmasking, grade the ease of ILM peel initiation, the downward force required and the ability to release the ILM from the forceps, using a 5-point scale ranging from 1 (very difficult) to 5 (very easy).

Surgical videos were graded by two independent observers (MF and AR) masked to the forceps and surgeon, with arbitration by a third (RJH) in cases of disagreement. Position and number of ILM primary and secondary pick-up points or attempts, any retinal haemorrhage, retinal trauma and ILM peeling duration were registered.

### Ophthalmic examination

A complete ophthalmic examination, including visual acuity (VA), slit-lamp biomicroscopy and dilated fundoscopy, was carried out preoperatively (within 14 days of surgery) and at 3 weeks, 3 and 6 months postoperatively. Visual acuity using a protocol refraction and ETDRS vision testing at 4 m was measured by masked research staff preoperatively and at 3 and 6 months postoperatively.

### Visual field protocol

Central VFs (Humphrey Field Analyzer Central 10–2 Swedish Interactive Threshold Algorithm-Standard test, Carl Zeiss Meditec, San Leandro, CA) were performed preoperatively and 6 months postoperatively. The test was performed in both eyes with the fellow eye first and repeated if they failed to achieve a pre-defined reliability criterion of < 15% false positives. Only VFs meeting these criteria were analysed. Technicians and assessors were masked to the forceps used.

### Imaging protocol and analysis

The Heidelberg Spectralis spectral-domain OCT device (Heidelberg Engineering, Heidelberg, Germany) was used by masked technicians. Posterior Pole scan, 30° × 25°, 240 Sects. (30 μm), high speed, ART 20 and a Peri-papillary Pre-set RNFL scan ART 100 were acquired preoperatively and at each FU. In addition, a 5° × 15°, 49 Sects. (30 μm), high speed, ART 20 was performed preoperatively and a separate high-definition infrared (IR) image with high ART (25) preoperatively and the 3-week visit. Images were exported anonymised for masked grading at a certified image grading centre (NetwORC UK, Central Angiographic Resource Facility, Queen’s University, Belfast, Northern Ireland).

**Fig. 2:**
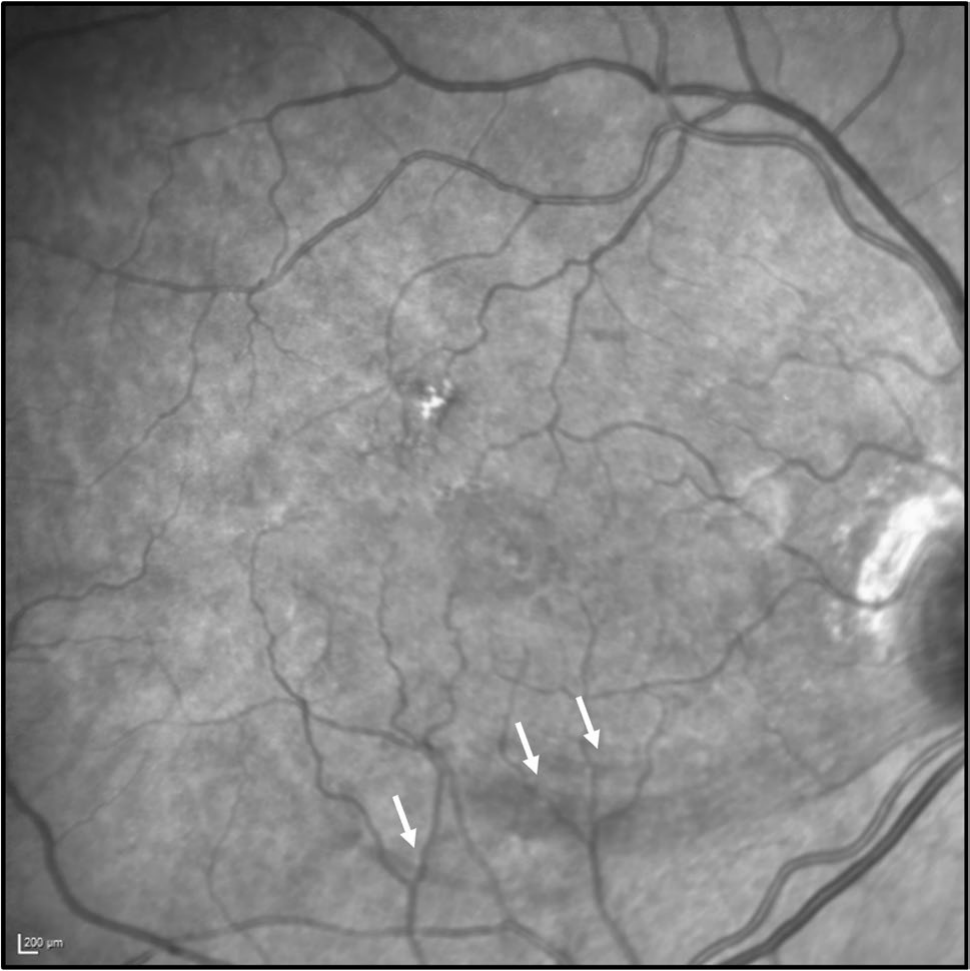
Three weeks postoperative infrared image illustrating a closed hole with 3 areas of subacute nerve fibre layer swelling shown as dark streaks inferior to the fovea (marked with white arrows)

The 3-week IR images were graded for the number of haemorrhages and dark RNFL lesions with corresponding RNFL thickening on SD-OCT representing SANFL lesions (Fig. 2), whereas 6-month images for external limiting membrane (ELM) or ellipsoid zone (EZ) defects and visible RNFL defects.

Using the Heidelberg auto-segmentation algorithm the inner retinal thickness (IRT), as a combined value of the RNFL, ganglion cell layer and inner plexiform layer thicknesses, was evaluated using the 1, 3, 6 mm ETDRS grid as well as the custom 8 × 8 grid centred on the fovea. The RNFL thickness was measured using the automated Heidelberg RNFL thickness algorithm in 6 peripapillary zones. All automatic segmentations were manually checked and adjusted in case of gross segmentation errors by masked graders.

**Fig. 3:**
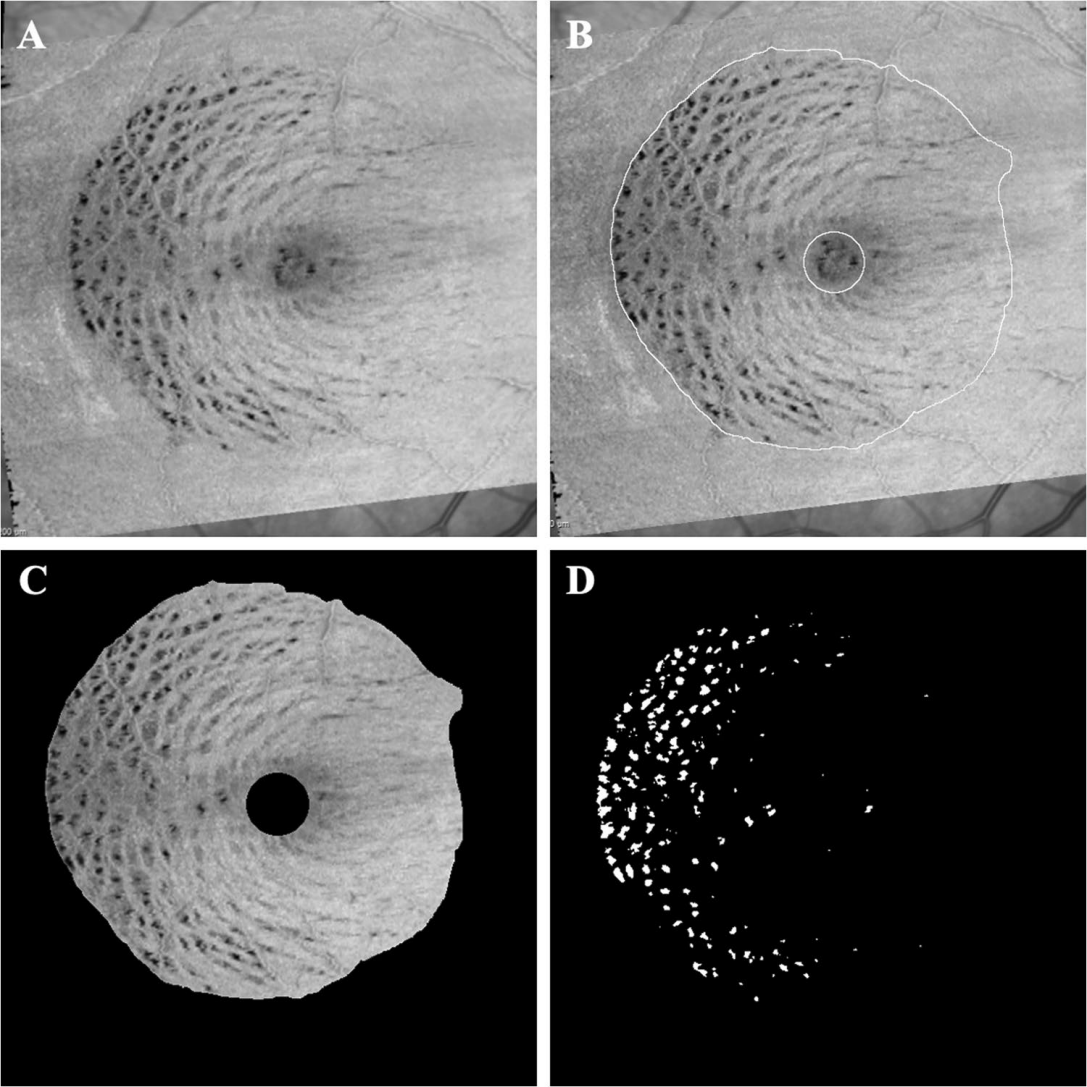
Composite diagram showing methodology for DONFL quantification. **A** Axial slab generated from the 6 months postoperative en-face OCT encompassing the inner surface of the ILM to outer border of the RNFL using the automated segmentation algorithm. **B** Slab imported into FIJI and scaled appropriately. Perimeter of ILM peel marked and mask placed over central 1000-micron diameter of foveal centre. **C** Area outside of peel and inside of foveal mask excluded. **D** After automatic thresholding, algorithm highlighting DONFL ‘dimples’

Finally, various parameters describing a dissociated optic nerve fibre layer (DONFL) appearance were calculated using the en-face OCT from the 6-month scans. An axial slab encompassing the inner surface of the ILM to the outer border of the RNFL using the automated segmentation algorithm was selected (Fig. 3A). This en-face image was then exported as a TIFF file and imported into FIJI, a biological-image analysis program [13, 14]. The images were converted to 8-bit images and had their scale set using the marker on each image. The ILM surgical peel outline was traced using the selection brush tool (Fig. 3B) to calculate the area of the ILM peeling. To represent the natural depression of the fovea, a circle of diameter 1000 µm centred at the foveal centre was placed (Fig. 3B). The area within this circle and outside of the ILM surgical peel was cleared and not used for the analysis (Fig. 3C). The automatic thresholding algorithm ‘MaxEntropy’ [14] was then applied to the remaining area by using the XOR function from the region of interest manager; this selected the DONFL ‘dimples’. The ImageJ ‘analyse particles’ function was used to obtain measurements for the number of distinct DONFL dimples (Fig. 3D).

### Statistical analysis

Due to randomisation, demographic and baseline characteristics were similar in the two groups, and thus, no formal statistical comparisons were made.

The remaining analyses compared 3-week and 6-month outcomes between the two study groups. Due to pandemic COVID restrictions, the 3-month imaging data was only available on 46/65 participants and hence was not analysed. Continuous variables normally and non-normally distributed were analysed using the unpaired *t*-test and the Mann–Whitney test, respectively. The Mann–Whitney test was used for the analysis of ordinal variables (e.g., ease of initiation of peel). Categorical variables were compared between groups using the chi-square test.

For VF data, only the index eyes’ data was used. The difference between the baseline and 6-month FU was used to compare the change in mean deviation (MD) between the two groups.

For the IRT data from the 8 × 8 grid data, the central 4 squares encompassing the foveal area and the peripheral squares (as variably outside the peel area) were excluded, meaning that only 32 pre-defined grid positions were included in the analysis. Inner retinal defects in these 32 grid positions were calculated and defined as follows. The baseline inner retina (IR) measurements were normalised in each square as follows:

$$\mathrm{Normalised}\;\mathrm{IR}=\mathrm{Original}\;\mathrm{IR}/\mathrm{mean}\;\mathrm{IR}\;\mathrm{fo}\;\mathrm{reach}\;\mathrm{patient}\times\mathrm{mean}\;\mathrm{IR}\;\mathrm{for}\;\mathrm{all}\;\mathrm{patients}$$

A ‘defect’ was defined as a value below 1.96 standard deviations below the mean using this normalised IR variable. Once the threshold was identified, for each patient, the number of defect areas was calculated.

## Results

Of 72 patients consented, 4 patients did not meet eligibility criteria, and 2 patients withdrew. The remaining 66 patients were randomised—33 patients in each group (Supplementary Fig. 1). One patient in the etched-tip group developed an intracranial haemorrhage before surgery and was withdrawn. The remaining 65 underwent surgery.

The groups were well-matched for baseline variables (Table 1). Sixty-two of the 65 (95.4%) patients had primary hole closure.

**Table 1:** Baseline variables

Variable	Etched-tip group	Smooth-tip group
Age, years (mean ± SD)	70.5 ± 6.7	70.0 ± 6.4
Male sex, *n* (%)	9 (27%)	9 (28%)
Axial length, millimetres (mean ± SD)	23.4 ± 1.0	23.2 ± 0.8
Previous/concurrent FTMH in fellow eye, *n* (%)	4 (12%)	6 (19%)
BCVA, ETDRS letters (mean ± SD)	50.1 ± 14.6	50.7 ± 9.2
Duration of symptoms, months (mean ± SD)	6.0 ± 2.4	6.0 ± 2.5
FTMH minimum linear diameter, μm (mean ± SD)	450 ± 172	473 ± 146
FTMH base diameter, μm (mean ± SD)	876 ± 315	919 ± 216
Presence of VMT, *n* (%)	9 (27%)	13 (41%)
Presence of ERM, *n* (%)	26 (79%)	26 (81%)
Presence of ERP, *n* (%)	3 (9%)	2 (6%)
Presence of VDA, *n* (%)	27 (82%)	28 (88%)
Pseudophakic at baseline, *n* (%)	9 (27%)	7 (22%)
Clinically significant cataract at baseline, *n* (%)	3 (9%)	6 (19%)

*BCVA*, best-corrected visual acuity; *ERM*, epiretinal membrane; *ERP*, epiretinal proliferation; *FTMH*, full-thickness macular hole; *SD*, standard deviation; *VDA*, vitreo-disc adhesion; *VMT*, vitreomacular traction

Three-week and 6-month follow-up were carried out for 65 and 63 patients, respectively, as 2 patients remained shielded due to COVID.

Three patients without primary closure underwent immediate repeat surgery using the randomised forceps type, with secondary closure, and were included in the 6-month analysis.

Four patients experienced ocular adverse events: 2 had pupillary optic capture requiring early revision surgery, and 2 had raised intraocular pressure postoperatively, resolved on topical treatment and normalised off drops at 6 months. There were no adverse events related to the forceps.

No surgeon declared that they were unmasked during the study as to the forceps type. The results of the surgeon questionnaire are summarised in Table 2. Despite the successful masking of forceps, there was a non-significant trend for the surgeons guessing the forceps type. Both forceps were judged to have similar performances.

**Table 2:** Results of surgeon questionnaire

Outcome	Category	Etched-tip *n* (%)	Smooth-tip *n* (%)	*p*-value
Guess	Etched-tip Smooth-tip Unsure	18 (55) 13 (39) 2 (2)	9 (28) 21 (66) 2 (6)	0.09
Correct guess	No* Yes	15 (45) 18 (55)	11 (34) 21 (66)	0.36
Ease of initiation of peel	Very difficult Difficult Neutral Easy Very easy	0 (0) 4 (12) 7 (21) 10 (30) 12 (36)	0 (0) 5 (16) 5 (16) 8 (25) 14 (44)	0.72
Ease of ILM grasping	Very difficult Difficult Neutral Easy Very easy	7 (21) 6 (18) 13 (39) 5 (15) 2 (6)	8 (25) 3 (9) 17 (53) 4 (13) 0 (0)	0.73
Ease of release	Very difficult Difficult Neutral Easy Very easy	0 (0) 3 (9) 6 (18) 10 (30) 14 (42)	0 (0) 0 (0) 8 (25) 13 (41) 11 (34)	0.87

^*^Unsure categorisation assumed to be incorrect

The comparison between parameters relating to the ILM peeling procedure is shown in Table 3. None of them differed significantly between the two groups.

**Table 3:** Internal limiting membrane peeling parameters

Variable	Etched-tip	Smooth-tip		*p*
	Mean ± SD	Mean ± SD	Mean (95% CI)	
Peel Area^*^	262 ± 67	264 ± 74	− 2 (− 41, 36)	0.91
Max peel diameter, μm	6526 ± 959	6581 ± 992	− 54 (− 588, 478)	0.84
Duration of peel, seconds	287 ± 130	303 ± 124	− 16 (− 98, 66)	0.70
	Median (IQR)	Median (IQR)	Median (95% CI)	
Retinal haemorrhages/case, number	1 (0, 1)	1 (0, 2)	− 1 (− 1, 0)	0.16
Pickup points attempts/case, number	3 (2, 5)	2 (2, 4)	1 (− 1, 2)	0.40
	*n* (%)	*n* (%)	% (95% CI)	
Whitening or deep retinal trauma (total number)	3 (14)	5 (29)	− 15 (− 41, 11)	0.26

*IQR*, interquartile range; *SD*, standard deviation; *CI*, confidence interval

^*^Peeling area summary values reported in 10^6^ μm

Primary pick-up points were located superiorly in 56% of cases, inferiorly in 25%, temporally in 12% and nasally in 7%.

On the 3-week images, there was no statistically significant difference in terms of the rate of eyes with SANFL lesions (63% vs 55%, *p* = 0.54) and retinal haemorrhages (16% vs 16%, *p* = 0.96) between the two groups. Moreover, the median number of SANFL lesion per eye did not significantly differ between the two groups (1 (IQR, 0, 1) in the etched-tip group and 1 (IQR, 0, 2) in the smooth-tip group, *p* = 0.75).

Regarding VFs, the mean values and differences of MD did not significantly differ between the two groups, varying from − 3.07 ± 4.09 preoperatively to − 1.97 ± 2.74 at 6-month follow-up (difference: 1.32 ± 3.59) in the etched-tip group and from − 2.61 ± 4.02 to − 2.24 ± 4.10 (difference: 1.14 ± 4.19) in the smooth-tip group.

Table 4 shows anatomical and functional outcomes at 6-month FU. The final VA, change in VA, number of DONFL lesions, presence of RNFL lesions, ELM, EZ and IR defects showed no significant differences between the groups. There was a non-significant trend towards a higher proportion of visible RNFL lesions in the etched-tip group.

**Table 4:** Anatomical and functional outcomes at 6-month follow-up

Outcome	Etched-tip	Smooth-tip		*p*
	Mean ± SD	Mean ± SD	Mean difference (95% CI)*	
BCVA, ETDRS letters	66.9 ± 11.2	70.9 ± 9.7	− 4.1 (− 9.4, 1.3)	0.13
Change in BCVA baseline to 6 months, ETDRS letters	15.0 ± 12.7	19.4 ± 8.2	− 4.4 (− 9.8, 1.1)	0.11
N. DONFL dimples	31.4 ± 22.2	41.0 ± 27.1	− 9.6 (− 23.1, 4.0)	0.16
	*N* eyes (%)	*N* eyes (%)	% (95% CI)	
ELM defects	5 (16)	7 (23)	− 7 (− 26, 12)	0.54
EZ defects	27 (84)	28 (90)	-6 (-22, 10)	0.48
Visible RNFL lesions	17 (53)	10 (32)	21 (− 3, 44)	0.09
N. inner retina defects				
None	20 (63)	24 (77)		
One Two Three	7 (22) 3 (9) 2 (6)	5 (16) 2 (6) 0 (0)		0.17

*BCVA*, best-corrected visual acuity; *DONFL*, dissociated optic nerve fibre layer; *ELM*, external limiting membrane; *EZ*, ellipsoid zone; *RNFL*, retinal nerve fibre layer

^*^Differences between groups reported as outcome for etched-tip group minus outcome for smooth-tip group

**Fig. 4:**
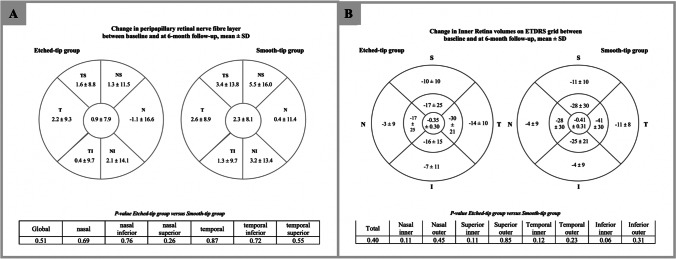
Composite diagram showing **A** changes in the peripapillary RNFL and **B** changes in inner retinal volume between preoperative and 6-month postoperative OCTs. *P* values shown in rows at base of each image

Regarding the changes in the RNFL parameters from baseline to 6 months, none of the peripapillary RNFL parameters varied significantly between the two groups (Fig. 4). The IR changes were similar in the two groups, except for a slight but not statistically significant, lower reduction in the inferior inner zone in the etched-tip group (Fig. 4).

## Discussion

This is the first proof-of-concept RCT assessing a newly developed types of ILM peeling forceps as compared with a standard one with a wide range of anatomical and functional endpoints. As a proof-of-concept study, it was successful, and the outcomes’ validity was strengthened by the trial design with robust randomisation and masking processes. Our trial provides a blue-print for future studies with outcome methodology and estimates that can be used to assess sample size to detect specific differences with appropriate sample sizes. Importantly, although RCT is a well-known tool for comparison, no previous study compared different types of ILM forceps, and no study design or set of outcomes has been previously validated to this aim.

We particularly focused on various endpoints to assess IR changes which occur consequently to ILM peeling and could be exacerbated by surgical trauma. The ILM is a < 10-μm thick, transparent membrane, forming the inner boundary of the retina. Although thin, its mechanical strength is in the megapascal range similar to articular cartilage and about 1000-fold stronger than the cellular layers, forming at least 50% of the retinal rigidity [15, 16].

Inner retinal layer changes occurring after ILM peeling can be focal related to direct instrument trauma or in a regular characteristic pattern as a consequence of Müller cell foot plate avulsion, termed DONFL [4, 17]. SANFL has been related to instrument trauma and detected as dark streaks on IR imaging in the early postoperative course associated with early focal NFL swelling on OCT and later focal IR atrophy. We graded these phenomena, as well as the extent of DONFL using en-face OCT and a variety of other retinal changes that has been described following FTMH surgery with ILM peeling [18]. We hypothesised that etched-tip forceps might reduce retinal trauma during ILM peeling and result in differences in the parameters evaluated, but we did not see any clear differences between the two forceps types. It is important however to again note that being a proof-of-concept study, the sample size was not chosen to assess differences in any one endpoint. There was a trend towards a lower amount of thinning of the paracentral retina in the etched-tip group that could be explored in future studies, but no clear trends in the measures of DONFL or SANFL.

It has been suggested that alternative ILM peeling techniques may be associated with varying degrees of retinal injury and recovery [19]. A differing extent of postoperative DONFL following surgery has been reported in a retrospective study comparing forceps to a diamond-dusted membrane scraper for ILM peeling in FTMH surgery [20, 21]. Similarly, a post-hoc analysis of eyes undergoing membrane peeling for vitreomacular interface disorders in the prospective PIONEER intraoperative OCT study showed that an acute post-peel increase in IRT was associated with the later development of DONFL appearance, suggesting that peeling angles and techniques may also influence this [22]. Various possible reasons may explain the absence of difference in any of the IR parameters we studied, aside from sample size. Surgeon experience may have reduced any potential surgeon-related differences. Furthermore, being masked to forceps type, the surgeons were not therefore able to alter their surgical approach based on forceps which could also affect results.

The functional consequences of the iatrogenic IR trauma and post-ILM peeling anatomical changes are uncertain. Some authors described paracentral scotomata, temporal VF defects and reduced retinal sensitivity after ILM peeling whilst others have not, and a DONFL appearance is not thought to have any functional consequences [23–25]. To assess visual function, we included visual acuity and central visual fields and did not find any difference in terms of final visual acuity and VF improvement in both groups.

We attempted to control for several other factors potentially related to IR changes. Vital dyes may influence the iatrogenic stress exerted on the retinal tissue [26], and dye toxicity might cause diffuse postoperative IR changes [27]. We therefore standardised the dye and intraoperative exposure using BBG, considered to have low toxicity [27]. The presence of ERM can deepen the cleavage plane of ILM removal resulting in greater degrees of Muller cell damage and, potentially, a greater extent of a DONFL appearance [17, 28]. We recorded the presence of ERM and epiretinal proliferation preoperatively, and the groups were well-matched for this. Surgeon experience may also influence retinal trauma [29]; therefore, we only included surgeons experienced in the pinch peeling technique and used a block randomisation system to ensure equal numbers of both forceps types were used by all surgeons.

There are several limitations to our study. Importantly, it was designed as a proof-of-concept study and therefore not powered to detect a specific level of difference between the two groups. Inter-surgeon variability could have masked clearer differences. Finally, we did not perform microperimetry and analysed the VFs via MD score; therefore, focal defects may have been missed. Assessing microperimetry at the grasp sites themselves could be analysed in future studies.

In conclusion, we successfully conducted a first-in-class masked randomised proof-of-concept study of two different forceps types used for ILM peeling. The findings do not support any clear benefit of etched-tip forceps over standard forceps during ILM peeling, but it is important to note that being a proof-of-concept study, the sample size was not chosen to assess any one endpoint. The trial design and data produced can serve as a model for future trials with appropriate sample sizes.

## Supplementary Information

Below is the link to the electronic supplementary material.

Supplementary Fig. 1 PRISMA flow diagram of trial (PNG 362 kb)

High resolution image (TIFF 766 kb)
