# Supplementary figures and images for: A randomised controlled trial evaluating internal limiting membrane peeling forceps in macular hole surgery
Source: Graefes Arch Clin Exp Ophthalmol. 2022 Dec 13;261(6):1553–62. doi: 10.1007/s00417-022-05932-y (PMC10198899; doi:10.1007/s00417-022-05932-y)

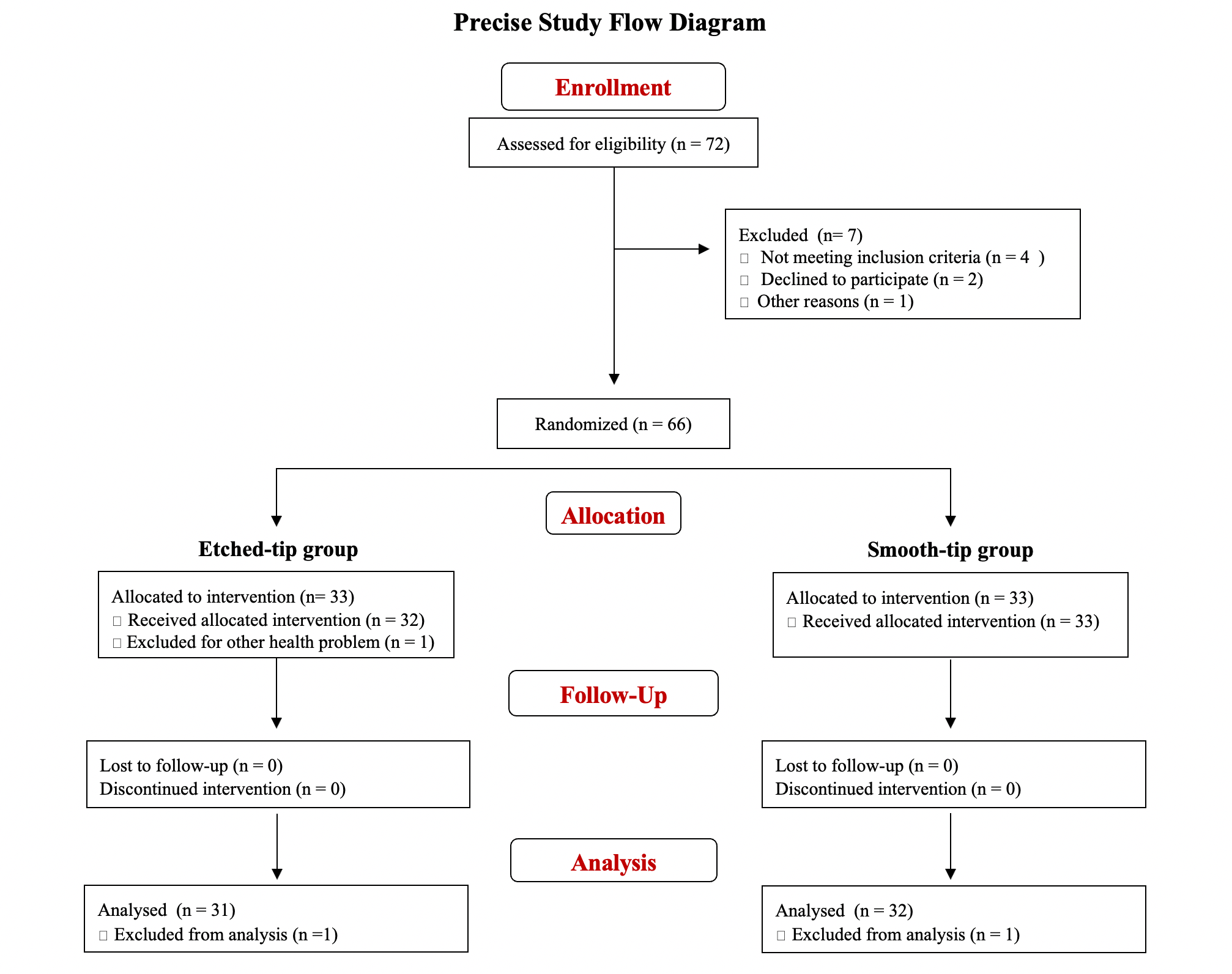

Supplement: Supplementary file 1 — PRISMA flow diagram of trial (PNG 362 kb) [file 417_2022_5932_Fig5_ESM.png]

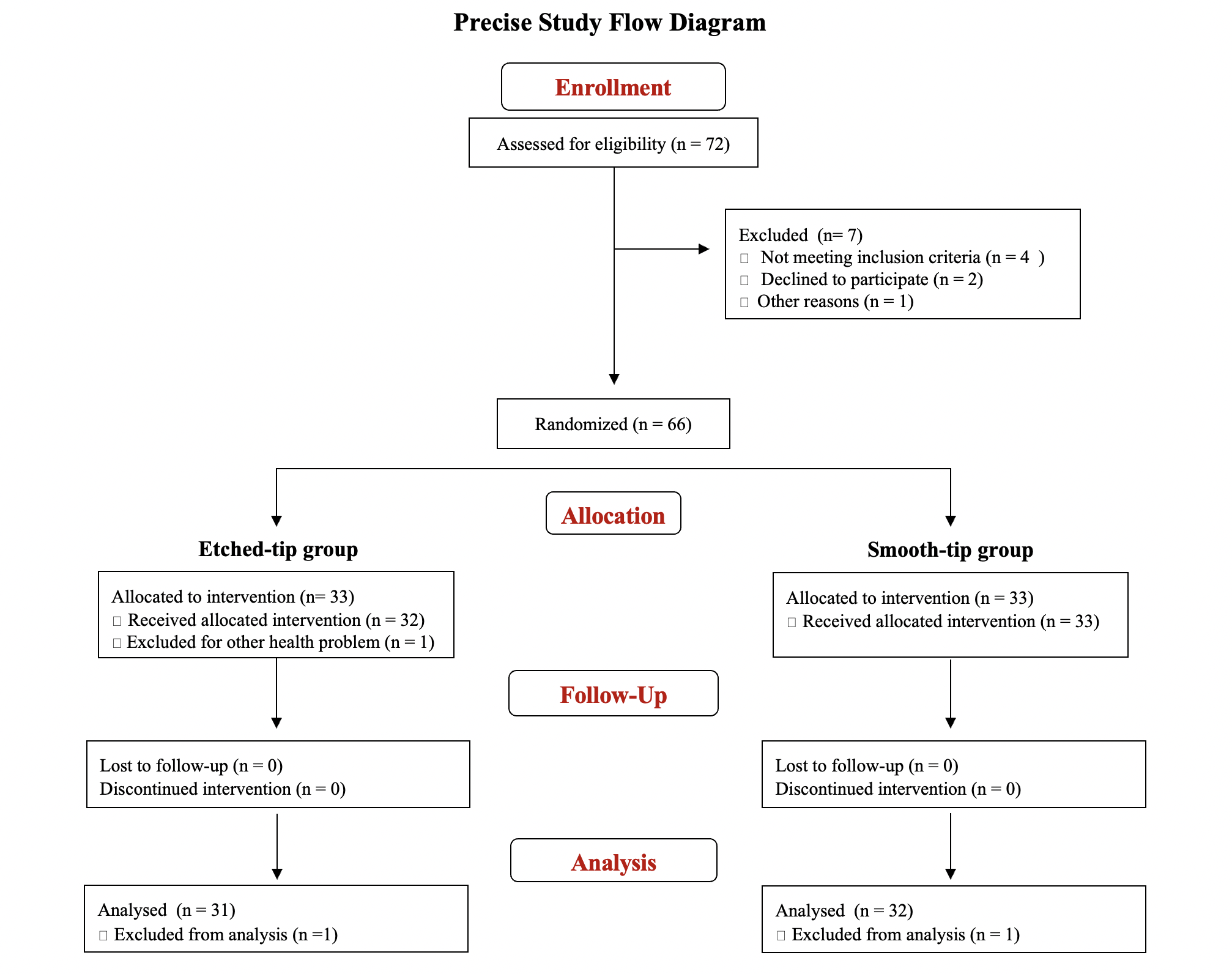

Supplement: Supplementary file 2 — High resolution image (TIFF 766 kb) [file 417_2022_5932_MOESM1_ESM.tiff]
